# Investigation of Enantioselective Membrane Permeability of α-Lipoic Acid in Caco-2 and MDCKII Cell

**DOI:** 10.3390/ijms17020155

**Published:** 2016-01-26

**Authors:** Ryota Uchida, Hinako Okamoto, Naoko Ikuta, Keiji Terao, Takashi Hirota

**Affiliations:** 1 Department of Biopharmaceutics, Faculty of Pharmaceutical Science, Tokyo University of Science, 2641 Yamazaki, Noda-shi, Chiba 278-8510, Japan; j3b13702@ed.tus.ac.jp; 2 CycloChem Bio Co., Ltd., KIBC654R 5-5-2 Minatojima-minamimachi Chuo-ku, Kobe 650-0047, Japan; hinako.okamoto@cyclochem.com (H.O.); keiji.terao@cyclochem.com (K.T.); 3 Graduate School of Medicine, Kobe University, 7-5-2 Kusunoki-cho Chuo-ku, Kobe 650-0017, Japan; naoko.ikuta@people.kobe-u.ac.jp

**Keywords:** α-lipoic acid, pharmacokinetics, enantioselective, membrane permeability, gastrointestinal availability, hepatic availability, Caco-2, MDCKII

## Abstract

α-Lipoic acid (LA) contains a chiral carbon and exists as two enantiomers (R-α-lipoic acid (RLA) and S-α-lipoic acid (SLA)). We previously demonstrated that oral bioavailability of RLA is better than that of SLA. This difference arose from the fraction absorbed multiplied by gastrointestinal availability (F_a_ × F_g_) and hepatic availability (F_h_) in the absorption phase. However, it remains unclear whether F_a_ and/or F_g_ are involved in enantioselectivity. In this study, Caco-2 cells and Madin–Darby canine kidney strain II cells were used to assess the enantioselectivity of membrane permeability. LA was actively transported from the apical side to basal side, regardless of the differences in its steric structure. Permeability rates were proportionally increased in the range of 10–250 µg LA/mL, and the permeability coefficient did not differ significantly between enantiomers. Hence, we conclude that enantioselective pharmacokinetics arose from the metabolism (F_h_ or F_g_ × F_h_), and definitely not from the membrane permeation (F_a_) in the absorption phase.

## 1. Introduction

α-Lipoic acid (LA; 5-(1,2-dithiolan-3-yl) pentanoic acid) is a sulfur-containing organic acid derived from octanoic acid. LA has attracted much attention because of its antioxidant and antidiabetic effects [1,2,3,4,5]. LA’s antioxidant properties are to scavenge reactive oxygen species (ROS) directly, to regenerate endogenous antioxidants, such as glutathione and vitamins E and C, and to reduce ROS production by metal-chelating. Moreover, LA plays a role as metabolic component of some enzymatic complexes involved in glucose metabolism of different cell types [6].

**Figure 1:**
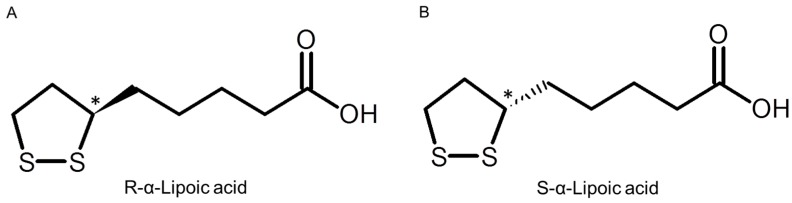
Structure of *R*-α-lipoic acid (**A**) and *S*-α-lipoic acid (**B**). Chiral center shown with asterisk (*).

LA has two sulfur atoms, one each at the C6 and C8 carbons. These sulfur atoms are combined by a disulfide bond, and LA exists as two enantiomers (Figure 1) since the C6 carbon is chiral. RLA and SLA seem to have different potencies. The RLA is more potent than the SLA to stimulate glucose uptake in L6 myotubes, and also to increase insulin-stimulated glucose uptake in obese Zucker rat [7]. In the body RLA is covalently bound to mitochondrial dehydrogenase enzyme complexes, which function as cofactors [8]. There is an increasing scientific and medical interest in potential therapeutic uses of LA [9]. In addition, enantioselective pharmacokinetics and pharmacodynamics of LA have been reported [10,11,12,13,14].

We have previously reported that the enantioselective pharmacokinetics of LA in rats was due to the fraction absorbed multiplied by gastrointestinal availability (F_a_ × F_g_) and hepatic availability (F_h_) in the absorption phase [15]. However, whether F_a_ and/or F_g_ were implicated in the enantioselectivity remains to be determined.

F_a_ or F_a_ × F_g_ can be calculated by the membrane permeability of drugs in the gastrointestinal tract, and for a long time, the membrane permeability has been estimated by measuring the permeation rate across a monolayer of Caco-2 cells [16,17]. Caco-2 cells express various nutrient transporters and are frequently used to analyze transporter functions [18,19]. Takaishi *et al.* [20] reported that the permeation of LA across the Caco-2 cell membrane may be mediated by some transporters. However, because they did not perform enantioselective measurements, namely, they measured LA concentration as a total of RLA and SLA, no information on the enantioselective transport was reported.

The aim of this study was to clarify whether membrane permeability in the absorption phase is enantioselective. We employed two cell types, Caco-2 cells and Madin–Darby canine kidney strain II (MDCKII) cells, which were recently used as a model of intestinal epithelial cells [21,22,23,24].

## 2. Results and Discussion

LA was reported to be transported by transporters such as monocarboxylate transporter (MCT) and sodium-dependent multivitamin transporter (SMVT) [20,25]. Caco-2 cells express these transporters [18], and MDCKII cells have recently been reported to express some uptake transporters [26,27]. Therefore, those cells were considered to represent an appropriate model to investigate whether membrane permeability of LA was enantioselective in the absorption phase. In this study, we measured the transport of LA enantiomers over the monolayers of Caco-2 and MDCKII cells.

**Figure 2:**
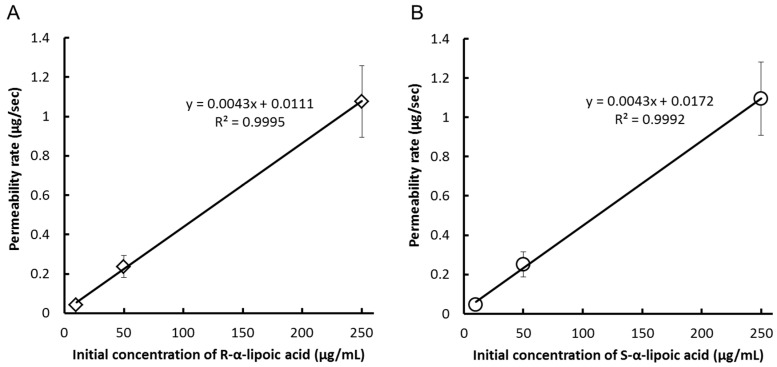
Correlation between initial concentrations *versus* permeability rate of (**A**) R-α-lipoic acid or (**B**) S-α-lipoic acid addition after addition of racemic α-lipoic acid to Caco-2 cell.

In Caco-2 cells, they were suitable for performing the transport experiment because the values of transepithelial electrical resistance (TER) were over 341–706 Ω/cm^2^, which was over the standard value (150 Ω/cm^2^) just before the experiments. The cumulative concentrations of each enantiomer were measured after the addition of the racemic mixture (mixture of equal parts of RLA-Na and SLA-Na) to the apical side of the cell monolayer (Table 1). Both enantiomers of LA were rapidly transported from the apical side to the basal side and were detected within 15 min. The permeability rate of LA in this study was about half of the previous reported results [20], which might be caused by a slight difference in experimental methods. However, overall results of the previous study and our present study were estimated to be not different. After 120 min, the concentration of LA was still higher on the apical than on the basal side in all groups. Significant difference in concentration in the basal side was observed only at two time point after 120 min (the low- and middle-concentration groups). However, we considered no enantiolectivity on the LA transport, because the difference was much smaller than that observed in our animal testing [15]. Permeability rates were linearly correlated (*R*^2^ > 0.9992) with the initial concentrations of LA at the apical side (Figure 2A,B), indicating that transport of LA across Caco-2 cells was not saturated in the concentration range used in this study. The apparent permeability (P_app_) did not differ significantly between the enantiomers at any concentration groups (Table 2). Therefore, we concluded that the transport of LA across Caco-2 cells was not enantioselective.

**Table 1:** The concentration-time profiles of α-lipoic acid after addition to the apical side of Caco-2 cell.

Side	Time (min)	Concentrations (µg/mL)					
		Low Group		Middle Group		High Group	
		RLA	SLA	RLA	SLA	RLA	SLA
basal	0	0	0	0	0	0	0
	15	0.06 ± 0.01	0.07 ± 0.01	0.36 ± 0.08	0.38 ± 0.10	1.61 ± 0.27	1.64 ± 0.28
	30	0.14 ± 0.01	0.15 ± 0.01	0.70 ± 0.09	0.73 ± 0.08	3.54 ± 0.39	3.66 ± 0.36
	60	0.27 ± 0.1	0.28 ± 0.01	1.28 ± 0.19	1.32 ± 0.20	7.09 ± 0.18	7.34 ± 0.14
	120	0.43 ± 0.02 *	0.46 ± 0.02	2.35 ± 0.12 *	2.47 ± 0.14	11.80 ± 0.38	12.19 ± 0.30
apical	120	1.94 ± 0.04	2.02 ± 0.05	10.64 ± 0.12	11.05 ± 0.28	65.01 ± 4.04	67.56 ± 3.47

Concentrations are shown as mean ± standard deviation (*n* = 3). LA, α-lipoic acid; RLA, R-α-lipoic acid; SLA, S-α-lipoic acid. * probability *p* < 0.01 compared with SLA. Statistical analysis was performed by using the paired-*t* test at each time point of each concentration group.

**Table 2:** Calculated P_app_ of Caco-2 cell by using the value of 15 min after addition of α-lipoic acid.

Cell Types	P_app_ (×10^−6^ cm/s)					
	Low Group		Middle Group		High Group	
	RLA	SLA	RLA	SLA	RLA	SLA
Caco-2	14.3 ± 2.2	15.4 ± 2.2	15.9 ± 3.7	16.7 ± 4.3	14.4 ± 2.4	14.6 ± 2.5

P_app_ are shown as mean ± standard deviation (*n* = 3). P_app_, apparent permeability; RLA, R-α-lipoic acid; SLA, S-α-lipoic acid. Statistical analysis was performed by using the paired-*t* test at each time point of each concentration group.

**Figure 3:**
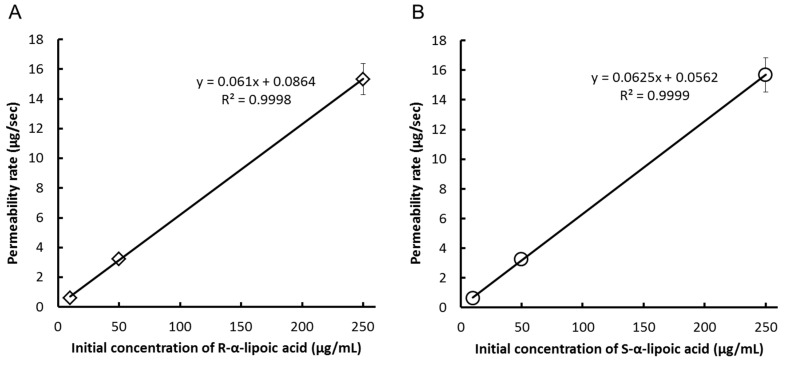
Correlation between initial concentration *versus* permeability rate of (**A**) R-α-lipoic acid or (**B**) S-α-lipoic acid addition after addition of α-lipoic acid to Mardin-Darby canine kidney II cell.

In MDCK II cells, they also were suitable for performing the transport experiment because the values of TER were over 228–248 Ω/cm^2^ which was over the standard value (150 Ω/cm^2^) just before the experiments. The cumulative concentrations of each enantiomer were measured after the addition of the racemic mixture to the apical side of the cell monolayer (Table 3). Although the concentration of LA detected at each time point were higher than that detected in Caco-2 cells experiments, LA was rapidly transported across the Caco-2 monolayer. After 120 min, the concentration of LA at the apical side was 1.4–3.6 times lower than that at the basal side in the all concentration groups (Low, Middle and High groups). These results suggest that LA was actively transported across the MDCK II monolayer. The correlation coefficient between the initial concentration at the apical side and permeability rates was determined using linear regression analysis (Figure 3A,B). Permeability rates were linearly correlated (*R*^2^ > 0.9998) with the initial concentrations of LA at the apical side. Hence, the transport of LA across MDCKII cells was not saturated in the concentration range used in this study. The P_app_ did not differ significantly between enantiomers at any concentration (Table 4). Therefore, we concluded that the transport of LA across MDCKII cells was not also enantioselective.

**Table 3:** The concentration-time profiles of α-lipoic acid after addition to the apical side of Mardin-Darby canine kidney II cell.

Side	Time (min)	Concentrations (µg/mL)					
		Low Group		Middle Group		High Group	
		RLA	SLA	RLA	SLA	RLA	SLA
basal	0	0	0	0	0	0	0
	15	0.90 ± 0.02	0.90 ± 0.03	4.90 ± 0.09	4.89 ± 0.10	23.00 ± 1.61	23.50 ± 1.65
	30	1.51 ± 0.03	1.52 ± 0.02	8.48 ± 0.57	8.57 ± 0.64	37.84 ± 2.21	38.79 ± 2.19
	60	2.33 ± 0.11	2.40 ± 0.12	12.82 ± 0.63	12.85 ± 0.68	58.44 ± 1.68	59.76 ± 1.73
	120	3.11 ± 0.17	3.04 ± 0.16	17.83 ± 1.20	17.74 ± 1.44	81.65 ± 2.68	83.69 ± 3.36
apical	120	0.86 ± 0.01	0.84 ± 0.02	6.02 ± 0.77	5.74 ± 0.62	59.23 ± 1.36	60.17 ± 1.46

Concentrations are shown as mean ± standard deviation (*n* = 3). LA, α-lipoic acid; RLA, R-α-lipoic acid; SLA, S-α-lipoic acid. Statistical analysis was performed by using the paired-*t* test at each time point of each concentration group.

Meanwhile, LA was transported across the monolayer of MDCKII much faster than that of Caco-2. The result was possibly derived from the difference in expression levels of transporters between both cells. Takaishi *et al*. [20] demonstrated the relationship between transporter expression and rac-LA transport using Caco-2 cells in 2007.

**Table 4:** Calculated P_app_ of Mardin-Darby canine kidney II cell by using the value of 15 min after addition of α-lipoic acid.

Cell Types	P_app_ (×10^−6^ cm/s)					
	Low Group		Middle Group		High Group	
	RLA	SLA	RLA	SLA	RLA	SLA
MDCK II	185.4 ± 5.5	186.2 ± 4.6	197.3 ± 2.6	197.7 ± 2.0	186.3 ± 12.7	189.8 ± 13.9

P_app_ are shown as mean ± standard deviation (*n* = 3). P_app_, apparent permeability; RLA, R-α-lipoic acid; SLA, S-α-lipoic acid; MDCK, Mardin-Darby canine kidney. Statistical analysis was performed by using the paired-*t* test at each time point of each concentration group.

We previously reported the enantioselective pharmacokinetics of LA in rats [15]. In that study, we observed that the F_h_ and F_a_ and/or F_g_ of transport from the gastrointestinal tract to the systemic circulation were implicated in these enantioselective pharmacokinetics. However, we could not determine whether F_a_ and/or F_g_ were responsible for this enantioselectivity. In this study, we found that the transport of LA across the monolayers of two cell types was not enantioselective. These results suggest that the enantioselective pharmacokinetics of LA arose from F_h_ or F_g_ × Fh, but not F_a_.

## 3. Experimental Section

### 3.1. Chemical and Reagents

RLA-Na (purity of >98.0%) was purchased from Toyo Hakko Co., Ltd. (Obu, Japan) and SLA-Na (purity of >85.0%) was supplied from Changshu Fushilai Medicine and Chemical Co., Ltd. (Changshu, China). Rac-LA (purity of >98.0%) and Dulbecco’s Modified Eagle Medium (DMEM) were purchased from Sigma-Aldrich Production GmbH (Buchs, Switzerland), and Rac-LA-d5 (purity of >98.0%) was purchased from Toronto Research Chemicals Inc. (Toronto, ON, Canada). Minimum essential medium (MEM), l-glutamine, penicillin-streptomycin, fetal bovine serum (FBS), and Hank’s balanced salt solution (HBSS) were purchased from ThermoFisher Science (Waltham, MA, USA). Antibiotic-Antimycotic Mixed Stock Solution (100×) was purchased from Nacalai Tesque Inc. (Kyoto, Japan). All other chemicals and reagents were commercially available and were of analytical grade or higher.

### 3.2. Cell Culture

#### 3.2.1. Caco-2

Caco-2 cells were obtained from DS Pharma Bio-medical (Osaka, Japan), and seeded at 4.0 × 10^5^ cells in 10 cm dishes. The cells were grown in 500 mL DMEM, Antibiotic-Antimycotic Mixed Stock Solution (100×), (5000 units/mL penicillin, 5000 µg/mL streptomycin and 12.5 µg/mL amphotericin B respectively), and 50 mL FBS. The cells were incubated at 37 °C in a humidified atmosphere of 5% CO_2_, and the medium was changed at 2–3 days intervals until cells reached confluence. Confluent cells (5–6 days after seeding) were plated at a cell density of 2.0 × 10^6^ per well in 24-well transwell plates and incubated until transport.

#### 3.2.2. MDCK II

MDCK II cells were obtained from Sumika Chemical Analysis Service, Ltd. (Osaka, Japan) and cultured at a density of 2.0 × 10^7^ cells in 175-cm^2^ plastic flasks. The culture medium comprised of the following reagents of the Gibco^®^ series (ThermoFisher Science): 500 mL MEM, 5 mL l-glutamine (200 mM), 5 mL penicillin-streptomycin (10,000 units/mL and 10,000 µg/mL, respectively), and 50 mL FBS. The cells were incubated at 37 °C in a humidified atmosphere of 5% CO_2_. Confluent cells (3–4 days after seeding) were plated at a cell density of 1.5 × 10^4^ per well in 24-well transwell plates and were incubated until transport.

### 3.3. Transport Experiments

#### 3.3.1. Caco-2

The medium was removed, and cells were washed with HBSS. To the apical side of the well, 500 µL of HBSS was added; to the basal side of the well, 1.0 mL of HBSS was added. The cells were incubated for 30 min at 37 °C, and the integrity of the cell layer was evaluated by measuring TER using Millicell^®^ (Millipore, Billerica, MA, USA). Then, the apical and basal solutions were removed, and 500 µL and 1.0 mL of the transport buffer (HBSS and 10 mM HEPES; pH 7.4) were added to the apical and basal sides of wells, respectively. After 30-min incubation, both solutions were removed; 600 µL of the receiver buffer (HBSS and 10 mM HEPES, pH 7.4, with 1% DMSO) was added to the basal side. LA was dissolved in HBSS (20, 100, and 500 µg/mL), and 150 µL of LA was added to the apical side of each well at room temperature. The upper final concentration in the assay, 500 µg/mL, was selected as the maximum concentration to be soluble in the buffer, and the following 2 concentrations were decided by 5-fold dilution in sequence. At 5, 15, 30, 60, and 120 min, samples were withdrawn from the basal chamber; then, they were diluted 2-fold in water, and stored at −20 °C until analysis.

#### 3.3.2. MDCK II

The medium was removed, and the cells were washed with HBSS. To the apical side of the well, 150 µL of HBSS was added; to the basal side, 600 µL of 4% bovine serum albumin (BSA)/HBSS was added. The cells were incubated for 15 min at 37 °C, and the integrity of the cell layer was evaluated as mentioned in above. Subsequently, the apical and basal solutions were removed, and 50 µL of HBSS and 600 µL of 4% BSA/HBSS were added into the apical and basal sides of the wells, respectively. One hundred microliters of LA solution was dissolved in HBSS (30, 150, and 750 µg/mL) and was added to the apical side of each well at room temperature. The final concentrations were same as those in Caco-2 experiments. Samples were taken and treated as mentioned above.

### 3.4. Determination of LA Concentration by LC-MS/MS

LC-MS/MS was performed using an API 3200^TM^ (AB SCIEX, Framingham, MA, USA) equipped with a Shimadzu Prominence HPLC system (Shimadzu, Kyoto, Japan) as previously described [15]. The HPLC system comprised CBM-20A system controller, a LC-20AD binary pump, DGU-20A^3^ degasser, SIL-20A autosampler and CTO-20A column oven. Briefly, each sample was mixed with four times its volume of acetonitrile containing 0.1% (*v/v*) formic acid and 200 ng/mL of *rac*-LA-d5 (internal standard). The mixture was centrifuged at 10,800× *g* and 4 °C for 10 min. The supernatant (10 μL) was applied to the LC-MS/MS system. The HPLC was performed using a CHIRALPAK AD-RH column (5 µm, 2.1 × 150 mm, Daicel, Osaka, Japan) at 30 °C with a mobile phase of 0.1% (*v/v*) formic acid/water (solvent A) and 0.1% (*v/v*) formic acid/methanol (solvent B). The analytes and internal standards were delivered at a flow rate of 0.3 mL/min. The proportion of solvent B in the mobile phase was held at 40% before the measurement. During the measurement, solvent B was increased linearly from 40% to 95% from 0 to 1.0 min, then hold the ratio of A/B at 5/95 until 6.0 min, and was decreased linearly from 95% to 40% during 0.1 min, then hold the ratio of A/B at 60/40 until 11.0 min. The analytes and internal standards eluted from the column were detected by the negative ion mode, and analyzed by multiple reaction monitoring mode of the transitions *m/z* 205.0 to 170.8 for *rac*-LA and *m/z* 210.0 to 173.8 for *rac*-LA-d5.

### 3.5. Data Analysis

Parameters are presented as the arithmetic mean ± standard deviation. Permeability rate was calculated using the following equation:

(Permeability rate) = *C*_t_ × V/t (1)

where *C*_t_ is the concentration of the basal solution at the time after the addition of LA (ng/mL), V is the volume of the basal solution (L), and t is the time after the addition of LA (sec). In this study the calculation was performed as *t* = 15 min. The proportionality of the permeability rate was calculated by linear regression. The permeability of LA enantiomers was measured as apparent permeability coefficients (P_app_) calculated using the following equation:

P_app_ = Permeability rate/*C*_0_/A (2)

where *C*_0_ is the initial concentration in the apical solution (ng/mL), and A is the surface area of the cells (cm^2^). P_app_ values of each enantiomer were compared using the paired-*t* tests. Differences were considered to be statistically significant at a *p* value of <0.01.

## 4. Conclusions

Membrane permeation of LA was not enantioselective in Caco-2 and MDCKII cell monolayers. The results of this study and our previous report suggest that the enantioselective pharmacokinetics of LA arose from F_h_ or F_g_ × Fh in the absorption phase.

## References

[1] Packer L., Witt E.H., Tritschler H.J. (1995). Α-lipoic acid as a biological antioxidant. Free Radic. Biol. Med..

[2] Biewenga G.P., Haenen G.R., Bast A. (1997). The pharmacology of the antioxidant lipoic acid. Gen. Pharmacol..

[3] Naito Y., Ikuta N., Okano A., Okamoto H., Nakata D., Terao K., Matsumoto K., Kajiwara N., Yasui H., Yoshikawa Y. (2015). Isomeric effects of anti-diabetic α-lipoic acid with gamma-cyclodextrin. Life Sci..

[4] Koriyama Y., Nakayama Y., Matsugo S., Kato S. (2013). Protective effect of lipoic acid against oxidative stress is mediated by Keap1/Nrf2-dependent heme oxygenase-1 induction in the RGC-5 cellline. Brain Res..

[5] Packer L., Kraemer K., Rimbach G. (2001). Molecular aspects of lipoic acid in the prevention of diabetes complications. Nutrition.

[6] Grasso S., Bramanti V., Tomassoni D., Bronzi D., Malfa G., Traini E., Napoli M., Renis M., Amenta F., Avola R. (2014). Effect of lipoic acid and α-glyceryl-phosphoryl-choline on astroglial cell proliferation and differentiation in primary culture. J. Neurosci. Res..

[7] Khanna S., Roy S., Packer L., Sen C.K. (1999). Cytokine-induced glucose uptake in skeletal muscle: Redox regulation and the role of α-lipoic acid. Am. J. Physiol..

[8] Bramanti V., Tomassoni D., Bronzi D., Grasso S., Curro M., Avitabile M., Li Volsi G., Renis M., Ientile R., Amenta F. (2010). Α-lipoic acid modulates GFAP, vimentin, nestin, cyclin D1 and MAP-kinase expression in astroglial cell cultures. Neurochem. Res..

[9] Kramer K., Packer L. (2001). R-α-lipoic acid. Oxid. Stress Dis..

[10] Niebch G., Buchele B., Blome J., Grieb S., Brandt G., Kampa P., Raffel H.H., Locher M., Borbe H.O., Nubert I. (1997). Enantioselective high-performance liquid chromatography assay of (+)*R*- and (−)*S*-α-lipoic acid in human plasma. Chirality.

[11] Hermann R., Niebch G., Borbe H.O., Fieger-Büschges H., Ruus P., Nowak H., Riethmüller-Winzen H., Peukert M., Blume H. (1996). Enantioselective pharmacokinetics and bioavailability of different racemic α-lipoic acid formulations in healthy volunteers. Eur. J. Pharm. Sci..

[12] Breithaupt-Grogler K., Niebch G., Schneider E., Erb K., Hermann R., Blume H.H., Schug B.S., Belz G.G. (1999). Dose-proportionality of oral thioctic acid—Coincidence of assessments via pooled plasma and individual data. Eur. J. Pharm. Sci..

[13] Streeper R.S., Henriksen E.J., Jacob S., Hokama J.Y., Fogt D.L., Tritschler H.J. (1997). Differential effects of lipoic acid stereoisomers on glucose metabolism in insulin-resistant skeletal muscle. Am. J. Physiol..

[14] Hagen T.M., Ingersoll R.T., Lykkesfeldt J., Liu J., Wehr C.M., Vinarsky V., Bartholomew J.C., Ames A.B. (1999). (*R*)-α-lipoic acid-supplemented old rats have improved mitochondrial function, decreased oxidative damage, and increased metabolic rate. FASEB J..

[15] Uchida R., Okamoto H., Ikuta N., Terao K., Hirota T. (2015). Enantioselective pharmacokinetics of α-lipoic acid in rats. Int. J. Mol. Sci..

[16] Artursson P., Karlsson J. (1991). Correlation between oral drug absorption in humans and apparent drug permeability coefficients in human intestinal epithelial (ca@Co-2) cells. Biochem. Biophys. Res. Commun..

[17] Yee S. (1997). *In vitro* permeability across Caco-2 cells (colonic) can predict *in vivo* (small intestinal) absorption in man—Fact or myth. Pharm. Res..

[18] Maubon N., le Vee M., Fossati L., Audry M., le Ferrec E., Bolze S., Fardel O. (2007). Analysis of drug transporter expression in human intestinal caco-2 cells by real-time PCR. Fundam. Clin. Pharmacol..

[19] Englund G., Rorsman F., Ronnblom A., Karlbom U., Lazorova L., Grasjo J., Kindmark A., Artursson P. (2006). Regional levels of drug transporters along the human intestinal tract: Co-expression of ABC and SLC transporters and comparison with Caco-2 cells. Eur. J. Pharm. Sci..

[20] Takaishi N., Yoshida K., Satsu H., Shimizu M. (2007). Transepithelial transport of α-lipoic acid across human intestinal Caco-2 cell monolayers. J. Agric. Food Chem..

[21] Irvine J.D., Takahashi L., Lockhart K., Cheong J., Tolan J.W., Selick H.E., Grove J.R. (1999). Mdck (Madin-Darby canine kidney) cells: A tool for membrane permeability screening. J. Pharm. Sci..

[22] Di L., Whitney-Pickett C., Umland J.P., Zhang H., Zhang X., Gebhard D.F., Lai Y., Federico J.J., Davidson R.E., Smith R. (2011). Development of a new permeability assay using low-efflux MDCKII cells. J. Pharm. Sci..

[23] Cho M.J., Thompson D.P., Cramer C.T., Vidmar T.J., Scieszka J.F. (1989). The madin darby canine kidney (MDCK) epithelial cell monolayer as a model cellular transport barrier. Pharm. Res..

[24] Volpe D.A. (2008). Variability in Caco-2 and mdck cell-based intestinal permeability assays. J. Pharm. Sci..

[25] Prasad P.D., Wang H., Huang W., Fei Y.J., Leibach F.H., Devoe L.D., Ganapathy V. (1999). Molecular and functional characterization of the intestinal Na^+^-dependent multivitamin transporter. Arch. Biochem. Biophys..

[26] Paroder V., Spencer S.R., Paroder M., Arango D., Schwartz S., Mariadason J.M., Augenlicht L.H., Eskandari S., Carrasco N. (2006). Na^+^/monocarboxylate transport (SMCT) protein expression correlates with survival in colon cancer: Molecular characterization of SMCT. Proc. Natl. Acad. Sci. USA.

[27] Pan G., Elmquist W.F. (2007). Mitoxantrone permeability in mdckii cells is influenced by active influx transport. Mol. Pharm..

